# Correction: Is Benin on track to reach universal household coverage of basic water, sanitation and hygiene services by 2030?

**DOI:** 10.1371/journal.pone.0335094

**Published:** 2025-10-21

**Authors:** The PLOS One Staff

## Notice of Republication

This article was republished on October 6, 2025, to correct an error in the last author’s name. The correct name is: Roger Salamon. The publisher apologizes for the errors. The correct citation is: Gaffan N, Dégbey C, Kpozèhouen A, Glèlè Ahanhanzo Y, Johnson RC, Salamon R (2023) Is Benin on track to reach universal household coverage of basic water, sanitation and hygiene services by 2030? PLoS One 18(5): e0286147. https://doi.org/10.1371/journal.pone.0286147

## Supporting information

S1 File Originally published, uncorrected article. (PDF)

S2 File Republished, corrected article. (PDF)
