# Supplementary material for: Is Benin on track to reach universal household coverage of basic water, sanitation and hygiene services by 2030?
Source: PLoS One. 2023 May 25;18(5):e0286147. doi: 10.1371/journal.pone.0286147 (PMC10212078; doi:10.1371/journal.pone.0286147)
Supplement: S1 Table — (PDF) [file pone.0286147.s001.pdf]

S1 Table. Basic household characteristics, Benin, 2001 to 2017-2018

Variables	DHS-II (2001)			DHS-III (2006)			DHS-IV (2011-2012)			DHS-V (2017-2018)			p
	n	%	95% CI	n	%	95% CI	n	%	95% CI	n	%	95% CI	
**Age (years)**													<0.001
<30	1158	20.14	18.81 - 21.54	3145	17.97	17.27 - 18.69	2571	14.81	14.16 - 15.47	2454	17.34	16.59 - 18.11	
30-39	1496	26.00	24.83 - 27.21	4955	28.31	27.50 - 29.13	4708	27.12	26.34 - 27.91	3810	26.91	26.04 - 27.81	
40-49	1118	19.44	18.35 - 20.57	3650	20.85	20.18 - 21.54	3609	20.78	20.12 - 21.46	2970	20.98	20.25 - 21.73	
50-59	785	13.64	12.69 - 14.65	2571	14.69	14.13 - 15.27	2955	17.02	16.41 - 17.64	2146	15.16	14.52 - 15.83	
≥60	1195	20.78	19.47 - 22.15	3182	18.18	17.46 - 18.92	3521	20.28	19.52 - 21.06	2775	19.60	18.74 - 20.50	
**Sex**													<0.001
Male	4566	79.15	77.60 - 80.63	13571	77.50	76.57 - 78.40	13427	77.07	76.10 - 78.00	10634	75.12	74.03 - 76.17	
Female	1203	20.85	19.37 - 22.40	3940	22.50	21.60 - 23.43	3995	22.93	22.00 - 23.90	3522	24.88	23.83 - 25.97	
**Level of education**													<0.001
No formal education	3259	57.18	54.78 - 59.54	9574	55.17	53.73 - 56.60	9268	54.53	53.12 - 55.93	7339	52.64	51.02 - 54.25	
Primary	1452	25.47	23.86 - 27.14	4310	24.84	23.91 - 25.79	3840	22.59	21.72 - 23.49	3232	23.18	22.25 - 24.13	
Secondary	828	14.53	13.23 - 15.93	2854	16.45	15.56 - 17.38	3003	17.67	16.83 - 18.54	2593	18.60	17.62 - 19.62	
Higher	161	2.83	2.16 - 3.69	615	3.55	3.15 - 3.99	885	5.21	4.65 - 5.83	778	5.58	4.96 - 6.28	
**Marital status**													<0.001
Single				3354	19.34	18.53 - 20.18	3837	22.04	21.16 - 22.93	3169	22.38	21.42 - 23.38	
In couple				13992	80.66	79.82 - 81.47	13578	77.96	77.07 - 78.84	10987	77.62	76.62 - 78.58	
**Wealth index**													0.360
Poorest				3473	19.83	18.46 - 21.28	3339	19.17	17.76 - 20.66	2501	17.67	16.02 - 19.45	
Poorer				3385	19.33	18.36 - 20.34	3286	18.86	17.91 - 19.85	2675	18.89	17.73 - 20.11	
Middle				3375	19.27	18.31 - 20.27	3346	19.21	18.23 - 20.22	2798	19.77	18.59 - 21.00	
Richer				3619	20.67	19.55 - 21.83	3569	20.49	19.31 - 21.72	2951	20.85	19.52 - 22.24	
Richest				3660	20.90	19.43 - 22.45	3881	22.28	20.90 - 23.72	3231	22.82	20.93 - 24.84	
**Household size**													0.017
≤5	3498	60.63	59.00 - 62.23	11012	62.88	61.88 - 63.87	11042	63.38	62.42 - 64.33	8728	61.66	60.40 - 62.90	
>5	2272	39.37	37.77 - 41.00	6499	37.12	36.13 - 38.12	6380	36.62	35.67 - 37.58	5428	38.34	37.10 - 39.60	
**CU5 in the household**													<0.001
No	2392	41.47	39.83 - 43.12	6954	39.71	38.73 - 40.70	7619	43.73	42.77 - 44.69	5598	39.55	38.39 - 40.72	
Yes	3377	58.53	56.88 - 60.17	10557	60.29	59.30 - 61.27	9803	56.27	55.31 - 57.23	8558	60.45	59.28 - 61.61	

n : weighted numbers by survey
% : weighted percentages by survey
95% CI : 95% Confidence Intervals of the weighted percentages by survey
p : p-value from the chi-2 test comparing the distribution of household characteristics between surveys

S1 Table. continued

Variables	DHS-II (2001)			DHS-III (2006)			DHS-IV (2011-2012)			DHS-V (2017-2018)			p
	n	%	95% CI	n	%	95% CI	n	%	95% CI	n	%	95% CI	
**Area**													<0.001
Urban	2147	37.21	35.56 - 38.90	7067	40.36	38.90 - 41.83	7698	44.18	43.01 - 45.37	6104	43.12	41.55 - 44.70	
Rural	3622	62.79	61.10 - 64.44	10444	59.64	58.17 - 61.10	9724	55.82	54.63 - 56.99	8052	56.88	55.30 - 58.45	
**Department**													<0.001
Alibori				1016	5.80	5.21 - 6.46	857	4.92	4.47 - 5.40	1192	8.42	7.59 - 9.32	
Atacora	669	11.60	10.41 - 12.90	1047	5.98	5.46 - 6.54	1251	7.18	6.47 - 7.96	923	6.52	5.80 - 7.32	
Atlantique	1346	23.34	21.93 - 24.81	2306	13.17	12.38 - 14.01	2358	13.53	12.84 - 14.26	1969	13.91	12.61 - 15.32	
Borgou	860	14.91	13.71 - 16.20	1416	8.09	7.34 - 8.90	1278	7.33	6.85 - 7.85	1498	10.58	9.74 - 11.48	
Collines				1411	8.06	7.50 - 8.66	1280	7.34	6.99 - 7.71	981	6.93	6.41 - 7.48	
Couffo				1266	7.23	6.67 - 7.82	1190	6.83	6.39 - 7.30	1108	7.83	7.18 - 8.52	
Donga				604	3.45	3.02 - 3.93	623	3.58	3.33 - 3.83	740	5.23	4.65 - 5.88	
Littoral				1887	10.78	9.92 - 11.69	2497	14.33	13.36 - 15.36	852	6.02	5.34 - 6.77	
Mono	765	13.26	12.19 - 14.42	1072	6.12	5.63 - 6.66	1071	6.15	5.77 - 6.55	879	6.21	5.66 - 6.81	
Ouémé	1015	17.59	16.20 - 19.08	2312	13.20	12.24 - 14.23	2192	12.58	11.76 - 13.46	1633	11.53	10.34 - 12.84	
Plateau				1032	5.89	5.55 - 6.26	1100	6.32	5.65 - 7.06	984	6.95	6.02 - 8.01	
Zou	1113	19.30	18.10 - 20.56	2142	12.23	10.61 - 14.07	1727	9.91	9.38 - 10.47	1399	9.88	9.24 - 10.55	
**Benin**	5769	100.00		17511	100.00		17422	100.00		14156	100.00		

n : weighted numbers by survey
% : weighted percentages by survey
95% CI : 95% Confidence Intervals of the weighted percentages by survey
p : p-value from the chi-2 test comparing the distribution of household characteristics between surveys
