# Supplementary material for: Is Benin on track to reach universal household coverage of basic water, sanitation and hygiene services by 2030?
Source: PLoS One. 2023 May 25;18(5):e0286147. doi: 10.1371/journal.pone.0286147 (PMC10212078; doi:10.1371/journal.pone.0286147)
Supplement: S2 Table — (PDF) [file pone.0286147.s002.pdf]

S2 Table. Level of household access to WASH services, Benin, 2001 to 2017-2018

Outcome variables	DHS-II (2001)			DHS-III (2006)			DHS-IV (2011-2012)			DHS-V (2017-2018)			p
	n	%	95% CI	n	%	95% CI	n	%	95% CI	n	%	95% CI	
**Basic water service**													<0.001
No	2853	49.46	45.83 - 53.11	6115	34.97	32.89 - 37.10	5102	29.28	27.53 - 31.10	5099	36.02	33.75 - 38.36	
Yes	2915	50.54	46.89 - 54.17	11373	65.03	62.90 - 67.11	12320	70.72	68.90 - 72.47	9057	63.98	61.64 - 66.25	
**Surface water**													<0.001
No	5160	89.46	86.13 - 92.06	15936	91.12	89.56 - 92.47	16786	96.35	95.34 - 97.15	13330	94.16	92.76 - 95.31	
Yes	608	10.54	7.94 - 13.87	1552	8.88	7.53 - 10.44	636	3.65	2.85 - 4.66	826	5.84	4.69 - 7.24	
**Basic sanitation service**													<0.001
No	5456	94.61	93.68 - 95.40	16478	94.23	93.57 - 94.83	14753	84.68	83.54 - 85.75	12274	86.71	85.41 - 87.91	
Yes	311	5.39	4.60 - 6.32	1009	5.77	5.17 - 6.43	2669	15.32	14.25 - 16.46	1882	13.29	12.09 - 14.59	
**Open defecation**													<0.001
No	1902	32.97	29.93 - 36.16	6682	38.21	36.21 - 40.26	7977	45.79	43.82 - 47.77	6525	46.09	43.54 - 48.67	
Yes	3866	67.03	63.84 - 70.07	10805	61.79	59.74 - 63.79	9445	54.21	52.23 - 56.18	7631	53.91	51.33 - 56.46	
**Basic hygiene service**													<0.001
No	5643	97.88	97.32 - 98.32	17022	97.95	97.59 - 98.25	15795	90.66	89.65 - 91.59	12725	89.89	88.86 - 90.84	
Yes	122	2.12	1.68 - 2.68	357	2.05	1.75 - 2.41	1627	9.34	8.41 - 10.35	1431	10.11	9.16 - 11.14	

n : weighted numbers by survey
% : weighted percentages by survey
95% CI : 95% Confidence Intervals of the weighted percentages by survey
p : p-value from chi-square test comparing the distribution of outcome variables between surveys
