# Supplementary material for: Is Benin on track to reach universal household coverage of basic water, sanitation and hygiene services by 2030?
Source: PLoS One. 2023 May 25;18(5):e0286147. doi: 10.1371/journal.pone.0286147 (PMC10212078; doi:10.1371/journal.pone.0286147)
Supplement: S3 Table — (PDF) [file pone.0286147.s003.pdf]

S3 Table. Association between household characteristics and access to basic drinking water services, Benin, 2001 to 2017-2018

Variables	DHS-II (2001)				DHS-III (2006)				DHS-IV (2011-2012)				DHS-V (2017-2018)			
	n	%	95% CI	p	n	%	95% CI	p	n	%	95% CI	p	n	%	95% CI	p
**Age (years)**				0.096				<0.001				<0.001				0.002
<30	618	53.37	48.88 - 57.80		2105	67.04	64.26 - 69.70		1858	72.28	69.73 - 74.70		1562	63.63	60.59 - 66.57	
30-39	775	51.84	47.70 - 55.95		3225	65.15	62.60 - 67.62		3378	71.74	69.57 - 73.81		2507	65.80	63.02 - 68.48	
40-49	548	49.03	44.66 - 53.41		2388	65.51	62.80 - 68.13		2593	71.85	69.65 - 73.95		1924	64.79	61.93 - 67.54	
50-59	396	50.42	45.48 - 55.35		1721	67.03	64.38 - 69.57		2063	69.81	67.46 - 72.05		1389	64.70	61.40 - 67.86	
≥60	572	47.89	42.84 - 52.97		1929	60.68	57.89 - 63.40		2385	67.72	65.10 - 70.24		1675	60.36	57.36 - 63.29	
**Sex**				0.016				<0.001				<0.001				<0.001
Male	2263	49.56	45.92 - 53.20		8646	63.78	61.61 - 65.89		9356	69.68	67.77 - 71.52		6654	62.57	60.20 - 64.88	
Female	652	54.24	49.25 - 59.15		2727	69.34	66.50 - 72.05		2964	74.19	71.84 - 76.41		2403	68.22	64.99 - 71.29	
**Level of education**				<0.001				<0.001				<0.001				<0.001
No formal education	1589	48.77	44.33 - 53.22		5414	56.62	54.13 - 59.08		5740	61.93	59.63 - 64.18		4148	56.52	53.82 - 59.18	
Primary	809	55.72	51.34 - 60.02		2978	69.19	66.50 - 71.76		2886	75.16	72.95 - 77.25		2164	66.97	63.90 - 69.90	
Secondary	447	54.00	49.51 - 58.43		2307	80.94	78.40 - 83.25		2499	83.20	81.01 - 85.19		1899	73.23	70.32 - 75.95	
Higher	38	23.47	. .		568	92.22	. .		838	94.69	. .		674	86.68	83.46 89.34	
**Marital status**								<0.001				0.001				0.019
Single					2378	70.95	68.45 - 73.33		2819	73.46	71.08 - 75.70		2098	66.20	63.04 - 69.21	
In couple					8894	63.66	61.43 - 65.83		9498	69.95	68.07 - 71.77		6959	63.34	61.00 - 65.62	
**Wealth index**								<0.001				<0.001				<0.001
Poorest					1456	41.98	38.75 - 45.28		1644	49.22	. .		1034	41.32	37.64 - 45.10	
Poorer					1806	53.43	50.15 - 56.68		2015	61.32	58.26 - 64.29		1412	52.78	49.36 - 56.17	
Middle					2052	60.92	57.93 - 63.83		2267	67.75	65.14 - 70.26		1653	59.06	55.71 - 62.34	
Richer					2736	75.76	73.08 - 78.25		2758	77.28	74.56 - 79.78		2086	70.68	67.48 - 73.70	
Richest					3322	90.81	88.76 - 92.52		3636	93.69	. .		2873	88.93	86.84 - 90.71	
**Household size**				0.174				<0.001				<0.001				<0.001
≤5	1798	51.43	47.42 - 55.43		7439	67.64	65.37 - 69.84		8026	72.69	70.79 - 74.51		5916	67.78	65.20 - 70.26	
>5	1117	49.16	45.22 - 53.10		3934	60.61	58.20 - 62.97		4294	67.30	65.11 - 69.43		3141	57.86	55.21 - 60.47	
**CU5 in the household**				0.043				<0.001				<0.001				<0.001
No	1166	48.73	44.78 - 52.70		4811	69.29	67.02 - 71.46		5542	72.75	70.79 - 74.62		3785	67.62	64.85 - 70.27	
Yes	1749	51.81	47.91 - 55.69		6562	62.23	59.92 - 64.49		6778	69.14	67.13 - 71.08		5271	61.60	59.19 - 63.95	

n : weighted numbers by survey
% : weighted percentages by survey
95% CI : 95% Confidence Intervals of the percentages by survey
p : for each survey, p-value from the chi-square test of the association between household characteristics and access to basic water services
. : missing standard errors because of stratum with single sampling unit

Table S3. continued

Variables	DHS-II (2001)				DHS-III (2006)				DHS-IV (2011-2012)				DHS-V (2017-2018)			
	n	%	95% CI	p	n	%	95% CI	p	n	%	95% CI	p	n	%	95% CI	p
**Area**				0.392				<0.001				<0.001				<0.001
Urban	1045	48.67	44.20 - 53.15		5608	79.41	76.81 - 81.78		6309	81.95	79.69 - 84.02		4474	73.30	70.01 - 76.35	
Rural	1870	51.64	46.44 - 56.81		5765	55.30	52.22 - 58.33		6011	61.82	59.11 - 64.46		4583	56.91	53.63 - 60.13	
**Department**				<0.001				<0.001				<0.001				<0.001
Alibori					540	53.14	44.22 - 61.85		453	52.85	45.39 - 60.19		426	35.76	29.28 - 42.81	
Atacora	232	34.68	27.42 - 42.72		444	42.43	36.22 - 48.89		702	56.11	50.45 - 61.62		469	50.78	43.77 - 57.75	
Atlantique	717	53.27	45.64 - 60.76		1473	63.88	56.76 - 70.44		1584	67.18	61.06 - 72.76		1385	70.36	62.34 - 77.30	
Borgou	356	41.40	33.77 - 49.46		946	66.79	57.90 - 74.63		842	65.87	58.70 - 72.38		788	52.63	46.14 - 59.03	
Collines					897	63.57	57.12 - 69.56		881	68.85	61.89 - 75.05		652	66.44	58.03 - 73.92	
Couffo					730	58.09	49.48 - 66.24		843	70.81	63.06 - 77.51		615	55.50	44.91 - 65.62	
Donga					233	38.68	30.56 - 47.48		348	55.92	46.84 - 64.62		316	42.67	33.14 - 52.79	
Littoral					1854	98.41	96.22 - 99.34		2397	96.02	94.57 - 97.09		839	98.54	96.98 - 99.31	
Mono	346	45.17	32.84 - 58.11		571	53.38	43.15 - 63.33		719	67.16	58.90 - 74.48		606	69.00	58.45 - 77.89	
Ouémé	554	54.56	45.22 - 63.60		1522	65.92	59.95 - 71.42		1631	74.42	67.99 - 79.93		1261	77.27	70.12 - 83.12	
Plateau					712	69.03	59.82 - 76.94		748	67.98	59.56 - 75.37		690	70.11	60.67 - 68.10	
Zou	711	63.84	54.64 - 72.12		1450	67.85	60.30 - 74.57		1172	67.90	61.38 - 73.79		1009	72.14	64.14 - 78.94	
**Benin**	2915	50.54	46.89 - 54.17		11373	65.03	62.90 - 67.11		12320	70.72	68.90 - 72.47		9057	63.98	61.64 - 66.25	

n : weighted numbers by survey
% : weighted percentages by survey
95% CI : 95% Confidence Intervals of the percentages by survey
p : for each survey, p-value from the chi-square test of the association between household characteristics and access to basic water services
