# Supplementary material for: Is Benin on track to reach universal household coverage of basic water, sanitation and hygiene services by 2030?
Source: PLoS One. 2023 May 25;18(5):e0286147. doi: 10.1371/journal.pone.0286147 (PMC10212078; doi:10.1371/journal.pone.0286147)
Supplement: S4 Table — (PDF) [file pone.0286147.s004.pdf]

S4 Table. Association between household characteristics and surface water consumption, Benin, 2001 to 2017-2018

Variables	DHS-II (2001)				DHS-III (2006)				DHS-IV (2011-2012)				DHS-V (2017-2018)			
	n	%	95% CI	p	n	%	95% CI	p	n	%	95% CI	p	n	%	95% CI	p
**Age (years)**				0.140				<0.001				0.107				0.099
<30	121	10.44	7.45 - 14.46		246	7.82	6.34 - 9.62		81	3.16	2.22 - 4.48		149	6.06	4.56 - 8.03	
30-39	148	9.88	7.29 - 13.27		432	8.72	7.24 - 10.48		174	3.70	2.78 - 4.92		193	5.05	3.92 - 6.50	
40-49	104	9.29	6.74 - 12.67		306	8.39	6.95 - 10.09		121	3.34	2.47 - 4.50		190	6.41	5.00 - 8.18	
50-59	82	10.44	7.17 - 14.96		211	8.22	6.57 - 10.25		104	3.52	2.64 - 4.68		117	5.45	4.22 - 7.02	
≥60	152	12.68	9.13 - 17.34		357	11.23	9.42 - 13.35		155	4.40	3.35 - 5.77		177	6.39	5.00 - 8.14	
**Sex**				0.001				<0.001				0.011				<0.001
Male	521	11.41	8.69 - 14.85		1341	9.89	8.37 - 11.65		524	3.90	3.01 - 5.03		700	6.58	5.30 - 8.15	
Female	87	7.23	4.72 - 10.92		212	5.38	4.24 - 6.80		112	2.81	2.08 - 3.79		126	3.59	2.59 - 4.95	
**Level of education**				<0.001				<0.001				<0.001				<0.001
No formal education	482	14.79	11.09 - 19.47		1225	12.81	10.90 - 15.00		503	5.43	4.23 - 6.94		614	8.37	6.75 - 10.34	
Primary	105	7.23	4.94 - 10.45		284	6.60	5.30 - 8.19		106	2.76	1.99 - 3.81		158	4.88	3.64 - 6.52	
Secondary	20	2.44	1.35 - 4.36		36	1.27	0.87 - 1.85		24	0.80	0.51 - 1.26		51	1.98	1.37 - 2.85	
Higher	0	0.00			1	0.19	. .		0	0.00			1	0.11	0.02 0.82	
**Marital status**								<0.001				0.002				0.005
Single					227	6.76	5.58 - 8.16		103	2.67	1.98 - 3.59		139	4.37	3.34 - 5.71	
In couple					1314	9.41	7.93 - 11.12		532	3.92	3.03 - 5.05		688	6.26	5.01 - 7.79	
**Wealth index**								<0.001				<0.001				<0.001
Poorest					872	25.14	21.78 - 28.83		412	12.35	. .		487	19.47	15.99 - 23.48	
Poorer					438	12.97	10.57 - 15.82		143	4.36	3.25 - 5.82		207	7.72	5.75 - 10.30	
Middle					186	5.53	4.42 - 6.90		72	2.14	1.48 - 3.08		97	3.47	2.54 - 4.72	
Richer					49	1.37	0.98 - 1.91		9	0.24	0.13 - 0.45		32	1.10	0.69 - 1.75	
Richest					6	0.17	0.08 - 0.34		0	0.00			3	0.10	0.03 - 0.32	
**Household size**				0.773				<0.001				0.371				0.011
≤5	374	10.70	7.75 - 14.58		844	7.67	6.43 - 9.13		389	3.52	2.72 - 4.55		454	5.21	4.09 - 6.61	
>5	234	10.30	7.69 - 13.67		709	10.92	9.18 - 12.93		247	3.87	2.92 - 5.11		372	6.85	5.37 - 8.69	
**CU5 in the household**				0.100				<0.001				0.018				0.038
No	229	9.59	6.93 - 13.12		515	7.42	6.14 - 8.93		248	3.25	2.50 - 4.23		290	5.17	4.03 - 6.61	
Yes	379	11.21	8.46 - 14.72		1038	9.84	8.33 - 11.58		388	3.96	3.06 - 5.11		537	6.27	5.00 - 7.84	

n : weighted numbers by survey
% : weighted percentages by survey
95% CI : 95% Confidence Intervals of the percentages by survey
p : for each survey, p-value from the chi-square test of the association between household characteristics and surface water consumption
. : missing standard errors because of stratum with single sampling unit

Table S4. continued

Variables	DHS-II (2001)				DHS-III (2006)				DHS-IV (2011-2012)				DHS-V (2017-2018)			
	n	%	95% CI	p	n	%	95% CI	p	n	%	95% CI	p	n	%	95% CI	p
**Area**				0.001				<0.001				<0.001				<0.001
Urban	111	5.17	3.08 8.57		228	3.22	2.10 4.92		50	0.65	0.35 1.18		164	2.69	1.76 - 4.10	
Rural	497	13.72	9.85 18.80		1325	12.71	10.61 15.14		586	6.03	4.62 7.82		662	8.22	6.38 - 10.52	
**Department**				0.020				<0.001				<0.001				<0.001
Alibori					69	6.74	3.84 - 11.56		6	0.72	0.25 - 2.03		39	3.27	1.66 - 6.36	
Atacora	135	20.14	12.67 - 30.47		273	26.04	19.93 - 33.24		71	5.70	3.33 - 9.59		78	8.40	4.88 - 14.08	
Atlantique	45	3.32	1.00 - 10.42		128	5.54	3.06 - 9.82		73	3.11	1.59 - 6.00		39	1.99	0.58 - 6.56	
Borgou	99	11.46	6.11 - 20.47		180	12.72	6.58 - 23.18		42	3.30	1.57 - 6.83		199	13.27	8.11 - 20.97	
Collines					88	6.22	3.51 - 10.81		24	1.89	0.69 - 5.05		60	6.11	2.50 - 14.21	
Couffo					72	5.73	2.54 - 12.43		28	2.39	0.76 - 7.29		61	5.50	2.20 - 13.09	
Donga					87	14.50	8.22 - 24.31		4	0.59	0.18 - 1.92		47	6.33	3.39 - 11.53	
Littoral					0	0.00			0	0.00			0	0.00		
Mono	87	11.41	4.60 - 25.59		49	4.61	1.67 - 12.09		19	1.75	0.52 - 5.70		26	2.90	0.81 - 9.85	
Ouémé	66	6.54	2.77 - 14.66		140	6.07	3.40 - 10.61		117	5.35	2.73 - 10.22		74	4.56	1.95 - 10.29	
Plateau					179	17.31	10.36 - 27.50		123	11.15	5.67 - 20.77		153	15.54	9.46 - 24.45	
Zou	176	15.85	8.30 - 28.14		288	13.48	8.43 - 20.85		128	7.40	3.96 - 13.40		51	3.66	1.20 - 10.58	
**Benin**	608	10.54	7.94 - 13.87		1552	8.88	7.53 - 10.44		636	3.65	2.85 - 4.66		826	5.84	4.69 - 7.24	

n : weighted numbers by survey

% : weighted percentages by survey

95% CI : 95% Confidence Intervals of the percentages by survey

p : for each survey, p-value from the chi-square test of the association between household characteristics and surface water consumption
