# Supplementary material for: Is Benin on track to reach universal household coverage of basic water, sanitation and hygiene services by 2030?
Source: PLoS One. 2023 May 25;18(5):e0286147. doi: 10.1371/journal.pone.0286147 (PMC10212078; doi:10.1371/journal.pone.0286147)
Supplement: S5 Table — (PDF) [file pone.0286147.s005.pdf]

S5 Table. Association between household characteristics and access to basic sanitation services, Benin, 2001 to 2017-2018

Variables	DHS-II (2001)				DHS-III (2006)				DHS-IV (2011-2012)				DHS-V (2017-2018)			
	n	%	95% CI	p	n	%	95% CI	p	n	%	95% CI	p	n	%	95% CI	p
**Age (years)**				<0.001				<0.001				<0.001				<0.001
<30	27	2.32	1.57 - 3.41		119	3.79	3.05 - 4.71		290	11.28	9.80 - 12.95		221	9.00	7.60 - 10.62	
30-39	68	4.57	3.46 - 6.01		241	4.87	4.15 - 5.70		657	13.96	12.41 - 15.67		462	12.12	10.58 - 13.84	
40-49	84	7.54	5.89 - 9.60		250	6.87	5.88 - 8.02		630	17.46	15.88 - 19.17		451	15.19	13.48 - 17.08	
50-59	68	8.66	6.65 - 11.19		207	8.07	6.85 - 9.47		527	17.83	16.05 - 19.74		356	16.60	14.39 - 19.07	
≥60	64	5.32	4.04 - 6.98		191	6.02	5.06 - 7.15		550	15.63	14.11 - 17.29		392	14.11	12.37 - 16.04	
**Sex**				0.080				0.087				0.190				0.140
Male	233	5.10	4.30 - 6.04		757	5.59	4.99 - 6.25		2025	15.08	13.96 - 16.28		1384	13.02	11.79 - 14.35	
Female	78	6.49	5.00 - 8.38		252	6.40	5.42 - 7.55		644	16.12	14.55 - 17.82		497	14.12	12.48 - 15.93	
**Level of education**				<0.001				<0.001				<0.001				<0.001
No formal education	35	1.07	0.77 - 1.50		369	3.86	3.34 - 4.46		659	7.11	6.33 - 7.97		448	6.10	5.31 - 7.01	
Primary	77	5.29	4.20 - 6.66		274	6.36	5.43 - 7.43		560	14.59	13.17 - 16.14		427	13.21	11.65 - 14.93	
Secondary	112	13.56	11.33 - 16.16		294	10.32	8.85 - 12.01		866	28.85	26.52 - 31.29		588	22.67	20.44 - 25.06	
Higher	74	45.88	. .		63	10.20	. .		488	55.15	. .		374	48.09	43.51 52.70	
**Marital status**								0.928				0.008				0.762
Single					193	5.77	4.91 - 6.77		651	16.95	15.34 - 18.70		427	13.47	11.89 - 15.23	
In couple					812	5.81	5.19 - 6.50		2018	14.86	13.75 - 16.04		1455	13.24	11.98 - 14.61	
**Wealth index**								<0.001				<0.001				<0.001
Poorest					3	0.09	0.02 - 0.43		5	0.15	. .		3	0.10	0.02 - 0.50	
Poorer					35	1.04	0.70 - 1.56		63	1.90	1.22 - 2.95		23	0.85	0.52 - 1.39	
Middle					108	3.20	2.52 - 4.05		233	6.97	5.91 - 8.20		72	2.57	1.96 - 3.36	
Richer					388	10.73	9.32 - 12.32		606	16.97	15.38 - 18.69		412	13.96	12.33 - 15.75	
Richest					475	13.02	11.52 - 14.67		1762	45.41	. .		1372	42.47	39.82 - 45.17	
**Household size**				<0.001				0.007				0.844				0.316
≤5	146	4.16	3.40 - 5.09		586	5.33	4.67 - 6.06		1697	15.37	14.19 - 16.63		1185	13.58	12.24 - 15.03	
>5	165	7.29	6.08 - 8.71		423	6.53	5.71 - 7.45		972	15.23	13.90 - 16.66		697	12.83	11.42 - 14.39	
**CU5 in the household**				<0.001				0.003				<0.001				<0.001
No	169	7.06	5.87 - 8.47		452	6.51	5.72 - 7.39		1391	18.26	16.90 - 19.72		895	15.98	14.46 - 17.63	
Yes	142	4.21	3.37 - 5.24		557	5.29	4.65 - 6.01		1277	13.03	11.94 - 14.20		987	11.53	10.29 - 12.90	

n : weighted numbers by survey
% : weighted percentages by survey
95% CI : 95% Confidence Intervals of the percentages by survey
p : for each survey, p-value from the chi-square test of the association between household characteristics and access to basic sanitation services
. : missing standard errors because of stratum with single sampling unit

Table S5. continued

Variables	DHS-II (2001)				DHS-III (2006)				DHS-IV (2011-2012)				DHS-V (2017-2018)			
	**n**	**%**	**95% CI**	**p**	**n**	**%**	**95% CI**	**p**	**n**	**%**	**95% CI**	**p**	**n**	**%**	**95% CI**	**p**
**Area**				<0.001				<0.001				<0.001				<0.001
Urban	269	12.54	10.61 - 14.75		619	8.77	7.61 - 10.08		2138	27.77	25.72 - 29.92		1365	22.37	20.08 - 24.83	
Rural	42	1.16	0.72 - 1.85		390	3.74	3.13 - 4.46		531	5.46	4.67 - 6.39		516	6.41	5.32 - 7.70	
**Department**				<0.001				<0.001				<0.001				<0.001
Alibori					25	2.47	1.46 - 4.16		18	2.10	1.28 - 3.40		47	3.97	2.47 - 6.30	
Atacora	5	0.72	0.30 - 1.73		24	2.25	1.29 - 3.90		64	5.13	2.61 - 9.83		39	4.20	2.10 - 8.22	
Atlantique	187	13.88	11.21 - 17.07		199	8.64	6.88 - 10.81		472	20.01	17.28 - 23.04		356	18.06	14.36 - 22.47	
Borgou	13	1.49	0.80 - 2.75		59	4.17	2.70 - 6.40		107	8.38	6.20 - 11.23		131	8.72	5.52 - 13.50	
Collines					30	2.12	1.27 - 3.53		147	11.46	8.30 - 15.63		51	5.19	3.41 - 7.82	
Couffo					40	3.16	2.18 - 4.58		68	5.75	3.95 - 8.30		62	5.62	3.64 - 8.57	
Donga					20	3.34	1.91 - 5.78		41	6.54	4.22 - 10.02		40	5.35	3.09 - 9.09	
Littoral					54	2.85	1.97 - 4.13		925	37.06	33.34 - 40.94		293	34.41	29.93 - 39.17	
Mono	21	2.79	1.38 - 5.58		38	3.52	2.15 - 5.72		70	6.53	4.53 - 9.34		92	10.48	7.25 - 14.91	
Ouémé	73	7.20	5.05 - 10.17		193	8.36	6.54 - 10.63		428	19.54	15.70 - 24.06		328	20.07	15.35 - 25.80	
Plateau					118	11.42	7.79 - 16.43		92	8.38	5.93 - 11.71		125	12.68	8.54 - 18.42	
Zou	12	1.09	0.51 - 2.32		210	9.83	7.46 - 12.86		236	13.69	10.26 - 18.03		319	22.81	17.30 - 29.46	
**Benin**	311	5.39	4.60 - 6.32		1009	5.77	5.17 - 6.43		2669	15.32	14.25 - 16.46		1882	13.29	12.09 - 14.59	

n : weighted numbers by survey
% : weighted percentages by survey
95% CI : 95% Confidence Intervals of the percentages by survey
p : for each survey, p-value from the chi-square test of the association between household characteristics and access to basic sanitation services
