# Supplementary material for: Is Benin on track to reach universal household coverage of basic water, sanitation and hygiene services by 2030?
Source: PLoS One. 2023 May 25;18(5):e0286147. doi: 10.1371/journal.pone.0286147 (PMC10212078; doi:10.1371/journal.pone.0286147)
Supplement: S6 Table — (PDF) [file pone.0286147.s006.pdf]

S6 Table. Association between household characteristics and open defecation, Benin, 2001 to 2017-2018

Variables	DHS-II (2001)				DHS-III (2006)				DHS-IV (2011-2012)				DHS-V 2017-2018			
	n	%	95% CI	p	n	%	95% CI	p	n	%	95% CI	p	n	%	95% CI	p
**Age (years)**				<0.001				<0.001				<0.001				<0.001
<30	804	69.37	65.05 - 73.38		1884	60.03	57.26 - 62.74		1361	52.96	49.85 - 56.04		1281	52.17	48.84 - 55.48	
30-39	944	63.17	59.08 - 67.08		2970	59.98	57.49 - 62.43		2455	52.15	49.68 - 54.61		1989	52.21	49.12 - 55.29	
40-49	701	62.74	58.58 - 66.72		2165	59.44	56.97 - 61.86		1876	51.98	49.49 - 54.46		1608	54.14	51.03 - 57.22	
s50-59	523	66.60	62.17 - 70.76		1565	60.91	58.08 - 63.67		1620	54.83	52.26 - 57.38		1129	52.59	49.29 - 55.87	
≥60	880	73.66	69.72 - 77.25		2214	69.71	67.21 - 72.09		2115	60.06	57.49 - 62.58		1625	58.55	55.41 - 61.61	
**Sex**				<0.001				<0.001				<0.001				<0.001
Male	3170	69.46	66.28 - 72.46		8652	63.84	61.74 - 65.88		7488	55.77	53.71 - 57.81		5928	55.74	53.05 - 58.40	
Female	695	57.80	52.99 - 62.47		2153	54.73	52.03 - 57.39		1957	48.97	46.32 - 51.64		1704	48.37	45.15 - 51.60	
**Level of education**				<0.001				<0.001				<0.001				<0.001
No formal education	2665	81.81	78.96 - 84.35		7509	78.56	76.74 - 80.26		6709	72.39	70.46 - 74.24		4991	68.01	65.43 - 70.49	
Primary	886	61.04	57.01 - 64.93		2335	54.22	51.64 - 56.79		1805	47.00	44.45 - 49.57		1572	48.65	45.33 - 51.98	
Secondary	283	34.16	29.36 - 39.31		828	29.06	26.58 - 31.67		784	26.11	23.88 - 28.48		913	35.22	32.16 - 38.41	
Higher	9	5.90	. .		36	5.89	. .		44	5.01	. .		86	11.00	8.68 13.86	
**Marital status**								<0.001				<0.001				0.001
Single					1874	55.94	53.22 - 58.62		1812	47.22	44.67 - 49.78		1599	50.46	47.23 - 53.68	
In couple					8828	63.18	61.05 - 65.27		7629	56.19	54.11 - 58.24		6032	54.90	52.25 - 57.53	
**Wealth index**								<0.001				<0.001				<0.001
Poorest					3453	99.41	99.04 - 99.64		3316	99.30	. .		2374	94.90	93.35 - 96.10	
Poorer					3086	91.24	89.13 - 92.96		3040	92.51	90.96 - 93.81		2171	81.18	78.06 - 83.95	
Middle					2653	78.82	76.56 - 80.91		2255	67.40	64.61 - 70.06		1883	67.31	64.02 - 70.44	
Richer					1377	38.13	35.57 - 40.75		804	22.53	20.35 - 24.87		1053	35.68	32.56 - 38.93	
Richest					236	6.46	5.41 - 7.68		29	0.76	. .		150	4.64	3.67 - 5.84	
**Household size**				0.715				<0.001				<0.001				<0.001
≤5	2337	66.81	63.37 - 70.08		6423	58.40	56.18 - 60.59		5605	50.76	48.61 - 52.92		4394	50.34	47.50 - 53.19	
>5	1529	67.36	63.71 - 70.81		4382	67.53	65.27 - 69.71		3839	60.18	57.90 - 62.41		3237	59.64	56.78 - 62.44	
**CU5 in the household**				<0.001				<0.001				<0.001				<0.001
No	1500	62.70	59.00 - 66.26		3854	55.51	53.17 - 57.82		3762	49.38	47.22 - 51.54		2770	49.49	46.49 - 52.49	
Yes	2366	70.10	66.77 - 73.22		6951	65.93	63.78 - 68.01		5683	57.96	55.80 - 60.10		4861	56.80	54.14 - 59.43	

n : weighted numbers by survey
% : weighted percentages by survey
95% CI : 95% Confidence Intervals of the percentages by survey
p : for each survey, p-value from the chi-square test of the association between household characteristics and open defecation
. : missing standard errors because of stratum with single sampling unit

Table S6. continued

Variables	DHS-II (2001)				DHS-III (2006)				DHS-IV (2011-2012)				DHS-V 2017-2018			
	n	%	95% CI	p	n	%	95% CI	p	n	%	95% CI	p	n	%	95% CI	p
**Area**				<0.001				<0.001				<0.001				<0.001
Urban	799	37.23	31.39 - 43.48		2312	32.76	29.45 - 36.25		1925	25.01	22.27 - 27.97		2093	34.29	30.22 - 38.60	
Rural	3067	84.69	81.14 - 87.67		8493	81.43	79.03 - 83.62		7519	77.33	74.96 - 79.53		5538	68.78	65.72 - 71.68	
**Department**				<0.001				<0.001				<0.001				<0.001
Alibori					901	88.67	81.20 - 93.41		733	85.49	78.97 - 90.24		793	66.54	57.42 - 74.57	
Atacora	607	90.74	84.65 - 94.57		934	89.28	83.57 - 93.17		1100	87.92	81.63 - 92.27		799	86.56	77.97 - 92.13	
Atlantique	559	41.54	34.78 - 48.63		1295	56.25	50.40 - 61.94		1061	45.01	39.46 - 50.68		739	37.51	29.58 - 46.17	
Borgou	673	78.35	71.36 - 84.02		1003	70.88	60.57 - 79.40		823	64.39	56.06 - 71.94		1053	70.31	61.59 - 77.76	
Collines					1170	82.97	78.18 - 86.89		919	71.84	64.60 - 78.11		699	71.25	62.88 - 78.39	
Couffo					931	73.66	65.66 - 80.35		875	73.51	66.25 - 79.69		727	65.61	56.95 - 73.34	
Donga					461	76.34	65.47 - 84.60		477	76.59	66.62 - 84.28		529	71.41	60.72 - 80.15	
Littoral					145	7.70	5.32 - 11.00		140	5.61	3.55 - 8.75		46	5.38	3.03 - 9.36	
Mono	558	73.00	62.02 - 81.74		759	71.11	62.60 - 78.35		646	60.34	52.22 - 67.92		496	56.46	46.12 - 66.27	
Ouémé	606	59.67	50.21 - 68.46		1167	50.60	44.09 - 57.09		979	44.65	38.15 - 51.34		633	38.80	29.60 - 48.86	
Plateau					712	69.03	61.27 - 75.84		659	59.86	50.58 - 68.48		583	59.27	48.76 - 68.99	
Zou	862	77.44	69.48 - 83.81		1326	61.97	54.78 - 68.66		1034	59.88	52.40 - 66.93		535	38.23	29.81 - 47.42	
**Benin**	3866	67.03	63.84 - 70.07		10805	61.79	59.74 - 63.79		9445	54.21	52.23 - 56.18		7631	53.91	51.33 - 56.46	

n : weighted numbers by survey
% : weighted percentages by survey
95% CI : 95% Confidence Intervals of the percentages by survey
p : for each survey, p-value from the chi-square test of the association between household characteristics and open defecation
