# Supplementary material for: Is Benin on track to reach universal household coverage of basic water, sanitation and hygiene services by 2030?
Source: PLoS One. 2023 May 25;18(5):e0286147. doi: 10.1371/journal.pone.0286147 (PMC10212078; doi:10.1371/journal.pone.0286147)
Supplement: S7 Table — (PDF) [file pone.0286147.s007.pdf]

S7 Table. Association between household characteristics and access to basic hygiene services, Benin, 2001 to 2017-2018

Variables	DHS-II (2001)				DHS-III (2006)				DHS-IV (2011-2012)				DHS-V (2017-2018)			
	n	%	95% CI	p	n	%	95% CI	p	n	%	95% CI	p	n	%	95% CI	p
**Age (years)**				0.001				0.061				0.091				0.226
<30	12	1.02	0.48 - 2.14		49	1.57	1.14 - 2.15		245	9.53	7.94 - 11.39		238	9.72	8.34 - 11.29	
30-39	34	2.26	1.57 - 3.23		97	1.98	1.59 - 2.47		449	9.54	8.19 - 11.09		380	9.98	8.62 - 11.52	
40-49	42	3.72	2.61 - 5.28		90	2.49	1.92 - 3.23		376	10.43	9.07 - 11.98		324	10.92	9.56 - 12.45	
50-59	21	2.68	1.66 - 4.29		64	2.49	1.85 - 3.35		268	9.07	7.81 - 10.51		234	10.92	9.24 - 12.87	
≥60	14	1.20	0.74 - 1.94		57	1.79	1.30 - 2.45		286	8.14	7.05 - 9.37		254	9.14	7.89 - 10.56	
**Sex**				0.641				0.581				0.160				0.556
Male	95	2.08	1.61 - 2.68		272	2.02	1.71 - 2.38		1230	9.16	8.20 - 10.21		1085	10.21	9.19 - 11.33	
Female	28	2.29	1.55 - 3.39		85	2.18	1.66 - 2.85		397	9.94	8.72 - 11.31		346	9.81	8.56 - 11.22	
**Level of education**				<0.001				<0.001				<0.001				<0.001
No formal education	5	0.16	0.06 - 0.38		39	0.41	0.28 - 0.58		477	5.15	4.42 - 5.99		437	5.95	5.22 - 6.77	
Primary	19	1.28	0.81 - 2.01		64	1.50	1.09 - 2.06		342	8.91	7.68 - 10.32		303	9.39	8.10 - 10.85	
Secondary	42	5.13	3.76 - 6.96		129	4.54	3.61 - 5.69		464	15.46	13.37 - 17.81		401	15.44	13.63 - 17.45	
Higher	52	32.39	. .		122	20.04	. .		272	30.68	. .		258	33.18	29.00 37.63	
**Marital status**								<0.001				0.230				0.551
Single					99	2.98	2.32 - 3.80		381	9.94	8.61 - 11.44		330	10.40	9.12 - 11.85	
In couple					258	1.86	1.55 - 2.22		1245	9.17	8.22 - 10.22		1101	10.02	9.03 - 11.11	
**Wealth index**								<0.001				<0.001				<0.001
Poorest					6	0.18	0.07 - 0.43		145	4.34	. .		96	3.82	2.92 - 4.98	
Poorer					5	0.14	0.06 - 0.34		153	4.67	3.61 - 6.02		146	5.47	4.44 - 6.73	
Middle					12	0.36	0.17 - 0.78		174	5.19	4.23 - 6.36		179	6.41	5.35 - 7.67	
Richer					23	0.65	0.36 - 1.18		309	8.67	7.43 - 10.10		255	8.63	7.39 - 10.05	
Richest					311	8.55	7.39 - 9.87		845	21.77	. .		755	23.37	20.79 - 26.17	
**Household size**				0.838				0.031				<0.001				0.033
≤5	75	2.15	1.65 - 2.80		245	2.24	1.89 - 2.65		1127	10.21	9.08 - 11.45		923	10.58	9.50 - 11.76	
>5	47	2.07	1.48 - 2.90		112	1.74	1.38 - 2.20		500	7.83	6.89 - 8.88		508	9.36	8.30 - 10.53	
**CU5 in the household**				<0.001				<0.001				<0.001				0.008
No	79	3.31	2.51 - 4.34		219	3.17	2.65 - 3.79		807	10.60	9.45 - 11.86		624	11.14	9.87 - 12.55	
Yes	43	1.28	0.92 - 1.78		138	1.32	1.06 - 1.64		819	8.36	7.42 - 9.40		807	9.44	8.45 - 10.53	

n : weighted numbers by survey
% : weighted percentages by survey
95% CI : 95% Confidence Intervals of the percentages by survey
p : for each survey, p-value from the chi-square test of the association between household characteristics and access to basic hygiene services
. : missing standard errors because of stratum with single sampling unit

Table S7. continued

Variables	DHS-II (2001)				DHS-III (2006)				DHS-IV(2011-2012)				DHS-V(2017-2018)			
	n	%	95% CI	p	n	%	95% CI	p	n	%	95% CI	p	n	%	95% CI	p
**Area**				<0.001				<0.001				<0.001				<0.001
Urban	113	5.26	4.11 - 6.71		306	4.36	3.73 - 5.11		1089	14.15	12.46 - 16.03		841	13.78	12.07 - 15.70	
Rural	10	0.26	0.12 - 0.59		50	0.49	0.28 - 0.85		537	5.53	4.67 - 6.53		590	7.32	6.32 - 8.48	
**Department**				<0.001				<0.001				<0.001				<0.001
Alibori					1	0.08	0.01 - 0.61		8	0.99	0.37 - 2.61		98	8.25	6.11 - 11.06	
Atacora	4	0.59	0.24 - 1.46		3	0.30	0.07 - 1.30		17	1.40	0.70 - 2.79		38	4.11	2.34 - 7.13	
Atlantique	81	6.05	4.50 - 8.10		30	1.30	0.77 - 2.20		271	11.50	8.63 - 15.16		359	18.24	13.75 - 23.78	
Borgou	3	0.36	0.12 - 1.13		14	0.99	0.49 - 1.97		150	11.73	9.62 - 14.24		178	11.90	9.50 - 14.81	
Collines					4	0.30	0.12 - 0.79		105	8.21	5.37 - 12.34		135	13.75	10.92 - 17.19	
Couffo					3	0.24	0.08 - 0.77		10	0.85	0.32 - 2.26		108	9.76	7.32 - 12.90	
Donga					1	0.14	0.02 - 1.05		11	1.71	0.98 - 2.98		41	5.52	3.33 - 9.04	
Littoral					203	10.84	9.06 - 12.93		479	19.17	15.64 - 23.27		246	28.83	23.80 - 34.44	
Mono	5	0.70	0.19 - 2.51		2	0.18	0.04 - 0.75		257	24.04	17.71 - 31.76		38	4.33	2.27 - 8.09	
Ouémé	23	2.22	1.16 - 4.21		68	2.98	1.80 - 4.88		203	9.24	7.22 - 11.75		121	7.44	5.45 - 10.07	
Plateau			-		16	1.60	0.76 - 3.33		66	5.99	3.92 - 9.05		15	1.50	0.78 - 2.88	
Zou	6	0.55	0.20 - 1.51		11	0.51	0.18 - 1.44		49	2.86	1.75 - 4.64		54	3.85	2.73 - 5.39	
Benin	122	2.12	1.68 - 2.68		357	2.05	1.75 - 2.41		1627	9.34	8.41 - 10.35		1431	10.11	9.16 - 11.14	

n: weighted numbers by survey
%: weighted percentages by survey
95% CI: 95% Confidence Intervals of the percentages by survey
p: for each survey, p-value from the chi-square test of the association between household characteristics and access to basic hygiene services
