# Supplementary material for: Is Benin on track to reach universal household coverage of basic water, sanitation and hygiene services by 2030?
Source: PLoS One. 2023 May 25;18(5):e0286147. doi: 10.1371/journal.pone.0286147 (PMC10212078; doi:10.1371/journal.pone.0286147)
Supplement: S8 Table — (PDF) [file pone.0286147.s008.pdf]

S8 Table. APCs of household access to basic drinking water services, Benin, 2001 to 2017-2018

Variables	APC			
	2001-2006	2006-2011	2012-2017	2001-2017
**Age (years)**				
<30	4.67	1.38	-2.10	1.07
30-39	4.68	1.77	-1.43	1.46
40-49	5.97	1.69	-1.71	1.70
50-59	5.86	0.74	-1.26	1.52
≥60	4.85	2.02	-1.90	1.41
**Sex**				
Male	5.18	1.62	-1.78	1.42
Female	5.03	1.24	-1.39	1.40
**Level of education**				
No formal education	3.03	1.64	-1.51	0.90
Primary	4.43	1.52	-1.90	1.12
Secondary	8.43	0.50	-2.10	1.86
Higher	31.48	0.48	-1.46	8.24
**Marital status**				
Single		0.63	-1.72	-0.60
In couple		1.73	-1.64	-0.04
**Wealth index**				
Poorest		2.93	-2.87	-0.14
Poorer		2.54	-2.47	-0.11
Middle		1.95	-2.26	-0.27
Richer		0.36	-1.48	-0.60
Richest		0.57	-0.87	-0.18
**Household size**				
≤5	5.63	1.32	-1.16	1.69
>5	4.28	1.92	-2.49	0.99
**CU5 in the household**				
No	7.29	0.89	-1.21	2.00
Yes	3.73	1.93	-1.91	1.05
**Area**				
Urban	10.29	0.58	-1.84	2.51
Rural	1.38	2.05	-1.37	0.59
**Department**				
Alibori	5.12	-0.10	-6.30	-0.88
Atacora	4.12	5.21	-1.65	2.34
Atlantique	3.70	0.92	0.77	1.70
Borgou	10.04	-0.25	-3.67	1.46
Collines	-0.08	1.46	-0.59	0.24
Couffo	5.16	3.66	-3.98	1.26
Donga	2.21	6.93	-4.41	1.27
Littoral	13.06	-0.45	0.43	3.80
Mono	3.40	4.26	0.45	2.60
Ouémé	3.85	2.23	0.63	2.13
Plateau	4.82	-0.28	0.52	1.53
Zou	1.23	0.01	1.01	0.74
**Benin**	5.17	1.53	-1.65	1.44
