# Supplementary material for: Is Benin on track to reach universal household coverage of basic water, sanitation and hygiene services by 2030?
Source: PLoS One. 2023 May 25;18(5):e0286147. doi: 10.1371/journal.pone.0286147 (PMC10212078; doi:10.1371/journal.pone.0286147)
Supplement: S9 Table — (PDF) [file pone.0286147.s009.pdf]

S9 Table. APCs of surface water consumption among households, Benin, 2001 to 2017-2018

Variables	APC			
	**2001-2006**	**2006-2011**	**2012-2017**	**2001-2017**
**Age (years)**	-5.61	-15.21	111.49	-3.24
<30	-2.47	-14.43	105.33	-3.98
30-39	-2.02	-15.41	111.47	-2.22
40-49	-4.67	-14.30	107.57	-3.86
50-59	-2.39	-15.66	106.41	-4.07
≥60				
**Sex**	-2.82	-15.57	109.11	-3.28
Male	-5.75	-11.15	104.18	-4.16
Female				
**Level of education**	-2.84	-14.46	107.49	-3.39
No formal education	-1.81	-14.68	110.00	-2.35
Primary	-12.26	-8.01	116.26	-1.26
Secondary		-100.00		-3.13
Higher				
**Marital status**		-15.52	108.55	-2.77
Single		-14.72	108.12	-2.59
In couple				
**Wealth index**		-12.13	107.89	-1.64
Poorest		-17.99	110.01	-3.29
Poorer		-15.86	108.39	-2.96
Middle		-27.00	128.66	-1.40
Richer		-100.00		-3.38
Richest				
**Household size**	-6.43	-13.19	106.73	-4.27
≤5	1.17	-17.20	110.00	-2.44
>5				
**CU5 in the household**	-5.01	-13.91	108.04	-3.67
No	-2.58	-15.27	107.98	-3.46
Yes				
**Area**	-9.03	-25.36	126.89	-3.88
Urban	-1.53	-12.68	105.31	-3.06
Rural				
**Department**				
Alibori	-10.06	-33.38	128.63	-7.31
Atacora	5.27	-24.14	106.67	-5.16
Atlantique	10.80	-9.95	92.80	-3.06
Borgou	2.12	-21.75	126.09	0.90
Collines	-17.06	-19.51	121.66	-5.61
Couffo	-12.86	-14.68	114.88	-4.32
Donga	-6.36	-44.20	148.68	-6.77
Littoral	-100.00			-100.00
Mono	-16.60	-16.14	108.80	-7.96
Ouémé	-1.46	-2.28	97.37	-2.16
Plateau	21.50	-7.69	105.68	5.39
Zou	-3.19	-10.32	88.93	-8.50
**Benin**	-3.38	-14.93	108.14	-3.52
