# Supplementary material for: Is Benin on track to reach universal household coverage of basic water, sanitation and hygiene services by 2030?
Source: PLoS One. 2023 May 25;18(5):e0286147. doi: 10.1371/journal.pone.0286147 (PMC10212078; doi:10.1371/journal.pone.0286147)
Supplement: S10 Table — (PDF) [file pone.0286147.s010.pdf]

S10 Table. APCs of household access to basic sanitation services, Benin, 2001 to 2017-2018

Variables	APC			
	2001-2006	2006-2011	2012-2017	2001-2017
**Age (years)**				
<30	10.33	21.90	-3.69	8.56
30-39	1.27	21.11	-2.33	6.08
40-49	-1.83	18.47	-2.29	4.34
50-59	-1.41	15.51	-1.18	4.02
≥60	2.51	18.94	-1.69	6.09
**Sex**				
Male	1.83	19.79	-2.42	5.84
Female	-0.26	18.27	-2.18	4.82
**Level of education**				
No formal education	29.17	11.73	-2.50	11.11
Primary	3.72	16.31	-1.65	5.70
Secondary	-5.31	20.54	-3.94	3.16
Higher	-25.97	35.92	-2.26	0.29
**Marital status**				
Single		21.64	-3.76	7.65
In couple		18.61	-1.91	7.42
**Wealth index**				
Poorest		9.21	-5.76	1.13
Poorer		11.56	-12.53	-1.73
Middle		15.21	-15.30	-1.88
Richer		8.70	-3.21	2.32
Richest		25.51	-1.11	10.83
**Household size**				
≤5	5.06	21.25	-2.05	7.43
>5	-2.18	16.66	-2.82	3.49
**CU5 in the household**				
No	-1.62	20.65	-2.20	5.08
Yes	4.66	17.82	-2.01	6.30
**Area**				
Urban	-6.90	23.32	-3.54	3.57
Rural	26.45	7.12	2.70	10.93
**Department**				
Alibori	10.67	-2.97	11.22	6.11
Atacora	25.66	16.13	-3.28	11.29
Atlantique	-9.04	16.49	-1.69	1.61
Borgou	22.85	13.53	0.66	11.30
Collines	14.19	35.90	-12.36	9.91
Couffo	2.55	11.46	-0.38	4.33
Donga	35.93	13.02	-3.31	12.93
Littoral	-27.11	59.38	-1.23	5.66
Mono	4.77	11.88	8.19	8.35
Ouémé	3.03	16.70	0.44	6.41
Plateau	9.66	-5.47	7.15	3.49
Zou	55.19	6.20	8.89	20.22
**Benin**	1.37	19.42	-2.34	5.62
