# Supplementary material for: Is Benin on track to reach universal household coverage of basic water, sanitation and hygiene services by 2030?
Source: PLoS One. 2023 May 25;18(5):e0286147. doi: 10.1371/journal.pone.0286147 (PMC10212078; doi:10.1371/journal.pone.0286147)
Supplement: S11 Table — (PDF) [file pone.0286147.s011.pdf]

S11 Table. APCs of open defecation among households, Benin, 2001 to 2017-2018

Variables	APC			
	2001-2006	2006-2011	2012-2017	2001-2017
**Age (years)**				
<30	-2.85	-2.25	-0.25	-1.71
30-39	-1.03	-2.51	0.02	-1.15
40-49	-1.08	-2.41	0.68	-0.89
50-59	-1.77	-1.89	-0.69	-1.42
≥60	-1.10	-2.67	-0.43	-1.38
**Sex**				
Male	-1.67	-2.43	-0.01	-1.32
Female	-1.09	-2.00	-0.21	-1.07
**Level of education**				
No formal education	-0.81	-1.47	-1.04	-1.11
Primary	-2.34	-2.56	0.58	-1.37
Secondary	-3.19	-1.93	5.11	0.18
Higher	-0.01	-2.89	14.00	3.86
**Marital status**				
Single		-3.03	1.11	-0.89
In couple		-2.11	-0.38	-1.21
**Wealth index**				
Poorest		-0.02	-0.75	-0.40
Poorer		0.25	-2.15	-1.01
Middle		-2.81	-0.02	-1.36
Richer		-9.12	7.96	-0.57
Richest		-32.26	35.23	-2.84
**Household size**				
≤5	-2.65	-2.52	-0.14	-1.70
>5	0.05	-2.07	-0.15	-0.74
**CU5 in the household**				
No	-2.41	-2.10	0.04	-1.42
Yes	-1.22	-2.31	-0.34	-1.27
**Area**				
Urban	-2.53	-4.79	5.40	-0.50
Rural	-0.78	-0.94	-1.93	-1.25
**Department**				
Alibori	2.50	-0.66	-4.09	-0.99
Atacora	-0.32	-0.28	-0.26	-0.29
Atlantique	6.25	-3.97	-2.99	-0.62
Borgou	-1.99	-1.73	1.48	-0.65
Collines	1.39	-2.58	-0.14	-0.50
Couffo	0.18	-0.04	-1.88	-0.65
Donga	-3.40	0.06	-1.16	-1.44
Littoral	-28.62	-5.59	-0.70	-11.66
Mono	-0.52	-2.94	-1.10	-1.54
Ouémé	-3.24	-2.25	-2.31	-2.58
Plateau	2.96	-2.56	-0.17	-0.04
Zou	-4.36	-0.62	-7.21	-4.19
**Benin**	-1.61	-2.35	-0.09	-1.31
