# Supplementary material for: Is Benin on track to reach universal household coverage of basic water, sanitation and hygiene services by 2030?
Source: PLoS One. 2023 May 25;18(5):e0286147. doi: 10.1371/journal.pone.0286147 (PMC10212078; doi:10.1371/journal.pone.0286147)
Supplement: S12 Table — (PDF) [file pone.0286147.s012.pdf]

S12 Table. APCs of household access to basic hygiene services, Benin, 2001 to 2017-2018

Variables	APC			
	2001-2006	2006-2011	2012-2017	2001-2017
**Age (years)**				
<30	9.04	38.85	0.32	14.66
30-39	-2.53	33.05	0.74	9.43
40-49	-7.71	29.75	0.77	6.75
50-59	-1.39	26.46	3.14	8.90
≥60	8.30	31.73	1.95	13.09
**Sex**				
Male	-0.59	31.66	1.83	10.13
Female	-1.03	31.81	-0.22	9.21
**Level of education**				
No formal education	21.26	58.65	2.45	24.74
Primary	3.30	38.22	0.87	12.85
Secondary	-2.41	24.97	-0.02	6.91
Higher	-9.15	8.05	1.31	0.15
**Marital status**				
Single		24.51	0.77	11.50
In couple		33.71	1.49	15.80
**Wealth index**				
Poorest		79.28	-2.11	30.74
Poorer		88.96	2.67	37.45
Middle		62.18	3.58	28.36
Richer		60.24	-0.08	25.24
Richest		18.53	1.19	9.14
**Household size**				
≤5	0.77	31.78	0.60	10.12
>5	-3.45	31.45	3.01	9.56
**CU5 in the household**				
No	-0.84	24.55	0.83	7.64
Yes	0.52	39.90	2.04	12.85
**Area**				
Urban	-3.65	23.85	-0.44	6.02
Rural	12.95	55.58	4.81	22.30
**Department**				
Alibori	-25.84	57.31	42.50	20.83
Atacora	-12.73	32.46	19.71	12.51
Atlantique	-26.43	48.56	7.99	6.91
Borgou	22.09	56.87	0.23	23.54
Collines	-11.29	82.28	8.99	21.55
Couffo	-19.06	25.69	50.11	17.33
Donga	-24.97	57.73	21.54	14.54
Littoral	12.37	10.92	7.04	9.92
Mono	-23.70	143.36	-24.85	11.69
Ouémé	6.02	22.87	-3.55	7.59
Plateau	-6.37	27.17	-20.58	-2.34
Zou	-1.30	36.57	5.07	12.51
**Benin**	-0.66	31.70	1.33	9.92
