# Supplementary material for: Is Benin on track to reach universal household coverage of basic water, sanitation and hygiene services by 2030?
Source: PLoS One. 2023 May 25;18(5):e0286147. doi: 10.1371/journal.pone.0286147 (PMC10212078; doi:10.1371/journal.pone.0286147)
Supplement: S13 Table — (PDF) [file pone.0286147.s013.pdf]

S13 Table. Projections of household access to basic drinking water services, Benin, 2019-2030

Variables	Projections (%)											
	**2019**	**2020**	**2021**	**2022**	**2023**	**2024**	**2025**	**2026**	**2027**	**2028**	**2029**	**2030**
**Age (years)**												
<30	64.66	65.35	66.05	66.76	67.47	68.20	68.93	69.67	70.41	71.17	71.93	72.70
30-39	67.24	68.22	69.22	70.22	71.25	72.28	73.34	74.40	75.49	76.59	77.70	78.83
40-49	66.45	67.58	68.73	69.90	71.10	72.31	73.54	74.79	76.07	77.36	78.68	80.02
50-59	66.18	67.19	68.21	69.25	70.31	71.38	72.46	73.57	74.69	75.82	76.98	78.15
≥60	61.65	62.52	63.40	64.30	65.21	66.13	67.06	68.01	68.97	69.95	70.93	71.94
**Sex**												
Male	63.91	64.82	65.75	66.68	67.63	68.59	69.57	70.56	71.56	72.58	73.62	74.66
Female	69.66	70.63	71.62	72.62	73.64	74.67	75.72	76.77	77.85	78.94	80.04	81.16
**Level of education**												
No formal education	57.28	57.80	58.31	58.84	59.37	59.90	60.44	60.98	61.53	62.08	62.64	63.20
Primary	68.10	68.86	69.63	70.41	71.20	72.00	72.81	73.62	74.45	75.28	76.13	76.98
Secondary	75.29	76.69	78.12	79.57	81.06	82.57	84.10	85.67	87.27	88.89	90.55	92.24
Higher	97.61	>99.00	>99.00	>99.00	>99.00	>99.00	>99.00	>99.00	>99.00	>99.00	>99.00	>99.00
**Marital status**												
Single	65.60	65.21	64.82	64.43	64.04	63.65	63.27	62.89	62.51	62.14	61.77	61.39
In couple	63.30	63.27	63.24	63.22	63.19	63.16	63.13	63.11	63.08	63.05	63.02	63.00
**Wealth index**												
Poorest	41.23	41.18	41.12	41.06	41.01	40.95	40.89	40.84	40.78	40.72	40.67	40.61
Poorer	52.69	52.64	52.58	52.53	52.47	52.42	52.36	52.31	52.25	52.19	52.14	52.08
Middle	58.83	58.67	58.51	58.35	58.20	58.04	57.89	57.73	57.58	57.42	57.27	57.11
Richer	70.05	69.63	69.21	68.79	68.38	67.97	67.56	67.15	66.75	66.35	65.95	65.55
Richest	88.68	88.52	88.36	88.20	88.04	87.88	87.72	87.56	87.40	87.24	87.08	86.92
**Household size**												
≤5	69.51	70.68	71.87	73.08	74.32	75.57	76.85	78.14	79.46	80.80	82.16	83.55
>5	58.73	59.31	59.90	60.49	61.09	61.70	62.31	62.93	63.56	64.19	64.82	65.47
**CU5 in the household**												
No	69.66	71.06	72.48	73.94	75.42	76.93	78.47	80.05	81.65	83.29	84.96	86.66
Yes	62.57	63.23	63.90	64.57	65.25	65.94	66.64	67.34	68.05	68.77	69.49	70.22
**Area**												
Urban	76.08	77.99	79.95	81.96	84.02	86.14	88.30	90.52	92.79	95.13	97.52	>99.00
Rural	57.42	57.76	58.10	58.44	58.79	59.13	59.48	59.83	60.19	60.54	60.90	61.26
**Department**												
Alibori	35.29	34.98	34.67	34.37	34.06	33.76	33.46	33.17	32.87	32.58	32.30	32.01
Atacora	52.57	53.80	55.05	56.34	57.66	59.01	60.39	61.80	63.24	64.72	66.24	67.78
Atlantique	72.16	73.39	74.64	75.91	77.20	78.51	79.84	81.20	82.58	83.99	85.41	86.87
Borgou	53.79	54.58	55.38	56.19	57.01	57.85	58.69	59.55	60.43	61.31	62.21	63.12
Collines	66.68	66.84	67.01	67.17	67.33	67.49	67.66	67.82	67.99	68.15	68.32	68.48
Couffo	56.55	57.26	57.98	58.71	59.45	60.20	60.96	61.72	62.50	63.28	64.08	64.88
Donga	43.49	44.04	44.59	45.16	45.73	46.31	46.90	47.49	48.09	48.70	49.32	49.94
Littoral	>99.00	>99.00	>99.00	>99.00	>99.00	>99.00	>99.00	>99.00	>99.00	>99.00	>99.00	>99.00
Mono	71.71	73.58	75.49	77.46	79.47	81.54	83.66	85.84	88.07	90.37	92.72	95.13
Ouémé	79.75	81.45	83.19	84.96	86.77	88.62	90.51	92.44	94.41	96.42	98.47	>99.00
Plateau	71.73	72.82	73.94	75.07	76.22	77.39	78.57	79.77	80.99	82.23	83.49	84.77
Zou	72.94	73.49	74.03	74.58	75.14	75.70	76.26	76.83	77.40	77.97	78.55	79.14
**Benin**	65.37	66.31	67.26	68.23	69.21	70.21	71.22	72.24	73.29	74.34	75.41	76.50
