# Supplementary material for: Is Benin on track to reach universal household coverage of basic water, sanitation and hygiene services by 2030?
Source: PLoS One. 2023 May 25;18(5):e0286147. doi: 10.1371/journal.pone.0286147 (PMC10212078; doi:10.1371/journal.pone.0286147)
Supplement: S14 Table — (PDF) [file pone.0286147.s014.pdf]

S14 Table. Projections of household surface water consumption, Benin, 2019-2030

Variables	Projections (%)											
	**2019**	**2020**	**2021**	**2022**	**2023**	**2024**	**2025**	**2026**	**2027**	**2028**	**2029**	**2030**
**Age (years)**												
<30	5.77	5.58	5.40	5.23	5.06	4.89	4.74	4.58	4.43	4.29	4.15	4.02
30-39	4.76	4.57	4.38	4.21	4.04	3.88	3.73	3.58	3.44	3.30	3.17	3.04
40-49	6.20	6.06	5.93	5.79	5.67	5.54	5.42	5.30	5.18	5.06	4.95	4.84
50-59	5.14	4.94	4.75	4.57	4.39	4.22	4.06	3.90	3.75	3.60	3.47	3.33
≥60	6.01	5.76	5.53	5.30	5.09	4.88	4.68	4.49	4.31	4.13	3.97	3.80
**Sex**												
Male	6.26	6.05	5.85	5.66	5.48	5.30	5.12	4.96	4.79	4.64	4.48	4.34
Female	3.37	3.23	3.09	2.97	2.84	2.72	2.61	2.50	2.40	2.30	2.20	2.11
**Level of education**												
No formal education	7.95	7.68	7.42	7.17	6.92	6.69	6.46	6.24	6.03	5.83	5.63	5.44
Primary	4.71	4.60	4.49	4.39	4.28	4.18	4.08	3.99	3.89	3.80	3.71	3.63
Secondary	1.94	1.91	1.89	1.87	1.84	1.82	1.80	1.77	1.75	1.73	1.71	1.69
Higher	<1.00	<1.00	<1.00	<1.00	<1.00	<1.00	<1.00	<1.00	<1.00	<1.00	<1.00	<1.00
**Marital status**												
Single	4.19	4.08	3.96	3.85	3.75	3.64	3.54	3.44	3.35	3.25	3.16	3.08
In couple	6.02	5.86	5.71	5.56	5.42	5.28	5.14	5.01	4.88	4.75	4.63	4.51
**Wealth index**												
Poorest	18.99	18.68	18.37	18.07	17.78	17.49	17.20	16.92	16.64	16.37	16.10	15.84
Poorer	7.35	7.10	6.87	6.64	6.43	6.21	6.01	5.81	5.62	5.44	5.26	5.09
Middle	3.32	3.22	3.12	3.03	2.94	2.85	2.77	2.69	2.61	2.53	2.45	2.38
Richer	1.08	1.06	1.05	1.03	1.02	1.00	<1.00	<1.00	<1.00	<1.00	<1.00	<1.00
Richest	<1.00	<1.00	<1.00	<1.00	<1.00	<1.00	<1.00	<1.00	<1.00	<1.00	<1.00	<1.00
**Household size**												
≤5	4.88	4.67	4.47	4.28	4.10	3.92	3.75	3.59	3.44	3.29	3.15	3.02
>5	6.60	6.44	6.28	6.13	5.98	5.83	5.69	5.55	5.41	5.28	5.15	5.03
**CU5 in the household**												
No	4.89	4.71	4.54	4.37	4.21	4.06	3.91	3.76	3.63	3.49	3.36	3.24
Yes	5.95	5.74	5.54	5.35	5.17	4.99	4.81	4.65	4.49	4.33	4.18	4.04
**Area**												
Urban	2.54	2.44	2.35	2.25	2.17	2.08	2.00	1.92	1.85	1.78	1.71	1.64
Rural	7.84	7.60	7.37	7.15	6.93	6.72	6.51	6.31	6.12	5.93	5.75	5.57
**Department**												
Alibori	2.92	2.71	2.51	2.32	2.15	2.00	1.85	1.72	1.59	1.47	1.37	1.27
Atacora	7.75	7.35	6.97	6.61	6.27	5.95	5.64	5.35	5.07	4.81	4.56	4.33
Atlantique	1.90	1.84	1.78	1.73	1.67	1.62	1.57	1.53	1.48	1.43	1.39	1.35
Borgou	13.45	13.57	13.69	13.82	13.94	14.06	14.19	14.32	14.45	14.58	14.71	14.84
Collines	5.61	5.29	5.00	4.72	4.45	4.20	3.97	3.74	3.53	3.34	3.15	2.97
Couffo	5.15	4.93	4.71	4.51	4.32	4.13	3.95	3.78	3.62	3.46	3.31	3.17
Donga	5.70	5.31	4.95	4.62	4.30	4.01	3.74	3.49	3.25	3.03	2.83	2.63
Littoral	<1.00	<1.00	<1.00	<1.00	<1.00	<1.00	<1.00	<1.00	<1.00	<1.00	<1.00	<1.00
Mono	2.56	2.36	2.17	2.00	1.84	1.69	1.56	1.43	1.32	1.21	1.12	1.03
Ouémé	4.41	4.32	4.22	4.13	4.04	3.96	3.87	3.79	3.70	3.62	3.55	3.47
Plateau	16.81	17.71	18.67	19.67	20.73	21.85	23.03	24.27	25.57	26.95	28.40	29.93
Zou	3.20	2.93	2.68	2.45	2.25	2.05	1.88	1.72	1.57	1.44	1.32	1.21
**Benin**	5.53	5.34	5.15	4.97	4.79	4.62	4.46	4.30	4.15	4.01	3.87	3.73
