# Supplementary material for: Is Benin on track to reach universal household coverage of basic water, sanitation and hygiene services by 2030?
Source: PLoS One. 2023 May 25;18(5):e0286147. doi: 10.1371/journal.pone.0286147 (PMC10212078; doi:10.1371/journal.pone.0286147)
Supplement: S15 Table — (PDF) [file pone.0286147.s015.pdf]

S15 Table. Projections of household access to basic sanitation services, Benin, 2019-2030

Variables	Projections (%)											
	2019	2020	2021	2022	2023	2024	2025	2026	2027	2028	2029	2030
**Age (years)**												
<30	10.18	11.05	12.00	13.02	14.14	15.35	16.66	18.09	19.64	21.32	23.14	25.12
30-39	13.24	14.04	14.90	15.81	16.77	17.79	18.87	20.02	21.24	22.53	23.90	25.35
40-49	16.19	16.89	17.63	18.39	19.19	20.02	20.89	21.80	22.74	23.73	24.76	25.83
50-59	17.61	18.32	19.06	19.82	20.62	21.45	22.31	23.21	24.15	25.12	26.13	27.18
≥60	15.42	16.36	17.35	18.41	19.53	20.72	21.98	23.32	24.74	26.25	27.84	29.54
**Sex**												
Male	14.17	15.00	15.88	16.81	17.79	18.83	19.93	21.09	22.32	23.62	25.00	26.47
Female	15.15	15.88	16.65	17.45	18.29	19.18	20.10	21.07	22.09	23.15	24.27	25.44
**Level of education**												
No formal education	7.15	7.94	8.83	9.81	10.90	12.11	13.45	14.94	16.60	18.45	20.50	22.78
Primary	14.35	15.17	16.03	16.94	17.91	18.93	20.01	21.15	22.35	23.62	24.97	26.39
Secondary	23.75	24.50	25.28	26.08	26.90	27.75	28.63	29.53	30.47	31.43	32.42	33.45
Higher	48.29	48.43	48.57	48.71	48.85	48.99	49.13	49.27	49.41	49.55	49.69	49.83
**Marital status**												
Single	15.05	16.20	17.44	18.77	20.21	21.76	23.42	25.21	27.14	29.22	31.45	33.86
In couple	14.74	15.83	17.01	18.27	19.63	21.08	22.65	24.33	26.14	28.08	30.16	32.40
**Wealth index**												
Poorest	<1.00	<1.00	<1.00	<1.00	<1.00	<1.00	<1.00	<1.00	<1.00	<1.00	<1.00	<1.00
Poorer	<1.00	<1.00	<1.00	<1.00	<1.00	<1.00	<1.00	<1.00	<1.00	<1.00	<1.00	<1.00
Middle	2.50	2.45	2.41	2.36	2.32	2.27	2.23	2.19	2.15	2.11	2.07	2.03
Richer	14.44	14.78	15.12	15.47	15.83	16.20	16.57	16.95	17.35	17.75	18.16	18.58
Richest	49.56	54.93	60.88	67.47	74.78	82.88	91.85	>99.00	>99.00	>99.00	>99.00	>99.00
**Household size**												
≤5	15.12	16.24	17.45	18.74	20.14	21.63	23.24	24.96	26.82	28.81	30.95	33.25
>5	13.51	13.98	14.47	14.98	15.50	16.04	16.60	17.18	17.78	18.40	19.04	19.70
**CU5 in the household**												
No	17.21	18.09	19.01	19.97	20.98	22.05	23.17	24.34	25.58	26.88	28.24	29.68
Yes	12.64	13.43	14.28	15.18	16.14	17.15	18.23	19.38	20.60	21.90	23.28	24.75
**Area**												
Urban	23.58	24.42	25.29	26.20	27.13	28.10	29.11	30.15	31.22	32.34	33.49	34.69
Rural	7.49	8.31	9.22	10.22	11.34	12.58	13.96	15.48	17.17	19.05	21.13	23.44
**Department**												
Alibori	4.33	4.60	4.88	5.18	5.49	5.83	6.19	6.56	6.97	7.39	7.84	8.32
Atacora	4.93	5.49	6.11	6.80	7.57	8.42	9.37	10.43	11.60	12.92	14.37	16.00
Atlantique	18.50	18.80	19.10	19.41	19.72	20.04	20.36	20.69	21.02	21.36	21.70	22.05
Borgou	10.23	11.39	12.68	14.11	15.70	17.48	19.45	21.65	24.10	26.82	29.85	33.22
Collines	5.98	6.58	7.23	7.94	8.73	9.60	10.55	11.59	12.74	14.00	15.39	16.92
Couffo	5.99	6.24	6.51	6.80	7.09	7.40	7.72	8.05	8.40	8.77	9.14	9.54
Donga	6.42	7.24	8.18	9.24	10.43	11.78	13.31	15.03	16.97	19.16	21.64	24.44
Littoral	37.37	39.48	41.71	44.07	46.56	49.20	51.98	54.92	58.03	61.31	64.78	68.44
Mono	11.82	12.81	13.88	15.03	16.29	17.65	19.12	20.72	22.45	24.32	26.35	28.55
Ouémé	22.03	23.45	24.95	26.55	28.25	30.06	31.99	34.04	36.22	38.55	41.02	43.65
Plateau	13.35	13.82	14.30	14.80	15.31	15.85	16.40	16.97	17.57	18.18	18.81	19.47
Zou	30.07	36.16	43.47	52.26	62.83	75.53	90.81	>99.00	>99.00	>99.00	>99.00	>99.00
**Benin**	14.43	15.24	16.10	17.00	17.96	18.96	20.03	21.16	22.35	23.60	24.93	26.33
