# Supplementary material for: Is Benin on track to reach universal household coverage of basic water, sanitation and hygiene services by 2030?
Source: PLoS One. 2023 May 25;18(5):e0286147. doi: 10.1371/journal.pone.0286147 (PMC10212078; doi:10.1371/journal.pone.0286147)
Supplement: S16 Table — (PDF) [file pone.0286147.s016.pdf]

S16 Table. Projections of open defecation among households, Benin, 2019-2030

Variables	Projections (%)											
	**2019**	**2020**	**2021**	**2022**	**2023**	**2024**	**2025**	**2026**	**2027**	**2028**	**2029**	**2030**
**Age (years)**												
<30	50.84	49.97	49.11	48.27	47.45	46.63	45.84	45.05	44.28	43.52	42.78	42.05
30-39	51.31	50.72	50.14	49.57	49.00	48.43	47.88	47.33	46.78	46.25	45.72	45.19
40-49	53.42	52.94	52.47	52.01	51.54	51.08	50.63	50.18	49.73	49.29	48.85	48.42
50-59	51.47	50.74	50.02	49.31	48.61	47.92	47.24	46.56	45.90	45.25	44.61	43.97
≥60	57.34	56.54	55.76	54.99	54.23	53.48	52.74	52.01	51.30	50.59	49.89	49.20
**Sex**												
Male	54.64	53.92	53.20	52.50	51.80	51.12	50.44	49.77	49.11	48.46	47.82	47.19
Female	47.59	47.08	46.57	46.07	45.58	45.09	44.61	44.13	43.65	43.18	42.72	42.26
**Level of education**												
No formal education	66.88	66.13	65.40	64.67	63.95	63.24	62.53	61.84	61.15	60.47	59.79	59.13
Primary	47.65	47.00	46.36	45.73	45.10	44.49	43.88	43.28	42.69	42.10	41.53	40.96
Secondary	35.32	35.38	35.45	35.51	35.58	35.64	35.71	35.78	35.84	35.91	35.97	36.04
Higher	11.65	12.10	12.56	13.05	13.55	14.07	14.61	15.18	15.76	16.37	17.00	17.66
**Marital status**												
Single	49.78	49.34	48.90	48.46	48.03	47.60	47.18	46.75	46.34	45.92	45.51	45.11
In couple	53.91	53.25	52.61	51.97	51.34	50.71	50.10	49.49	48.89	48.29	47.71	47.13
**Wealth index**												
Poorest	94.33	93.95	93.57	93.19	92.81	92.44	92.07	91.70	91.33	90.96	90.59	90.23
Poorer	79.96	79.15	78.35	77.56	76.77	76.00	75.23	74.47	73.72	72.97	72.24	71.51
Middle	65.94	65.04	64.15	63.28	62.41	61.56	60.72	59.90	59.08	58.28	57.48	56.70
Richer	35.38	35.17	34.97	34.77	34.57	34.37	34.18	33.98	33.78	33.59	33.40	33.21
Richest	4.44	4.32	4.19	4.07	3.96	3.85	3.74	3.63	3.53	3.43	3.33	3.24
**Household size**												
≤5	49.06	48.23	47.41	46.60	45.81	45.03	44.27	43.51	42.77	42.05	41.33	40.63
>5	58.99	58.55	58.12	57.69	57.27	56.85	56.43	56.02	55.60	55.20	54.79	54.39
**CU5 in the household**												
No	48.43	47.74	47.06	46.39	45.73	45.08	44.44	43.80	43.18	42.57	41.96	41.36
Yes	55.73	55.02	54.32	53.64	52.96	52.29	51.62	50.97	50.32	49.69	49.06	48.44
**Area**												
Urban	34.04	33.87	33.70	33.53	33.36	33.20	33.03	32.87	32.70	32.54	32.38	32.22
Rural	67.49	66.64	65.81	64.98	64.17	63.37	62.57	61.79	61.01	60.25	59.49	58.75
**Department**												
Alibori	65.56	64.91	64.27	63.64	63.01	62.39	61.78	61.17	60.57	59.97	59.38	58.79
Atacora	86.19	85.94	85.70	85.45	85.21	84.96	84.72	84.48	84.24	84.00	83.76	83.52
Atlantique	37.17	36.94	36.71	36.48	36.26	36.03	35.81	35.59	35.37	35.15	34.94	34.72
Borgou	69.62	69.16	68.71	68.26	67.81	67.37	66.93	66.49	66.06	65.62	65.19	64.77
Collines	70.72	70.36	70.01	69.65	69.30	68.95	68.61	68.26	67.92	67.58	67.24	66.90
Couffo	64.97	64.55	64.14	63.72	63.31	62.91	62.50	62.10	61.70	61.30	60.90	60.51
Donga	69.87	68.87	67.87	66.89	65.93	64.98	64.04	63.12	62.21	61.31	60.43	59.56
Littoral	4.46	3.94	3.48	3.08	2.72	2.40	2.12	1.88	1.66	1.46	1.29	1.14
Mono	55.16	54.31	53.47	52.64	51.83	51.03	50.24	49.46	48.70	47.95	47.21	46.48
Ouémé	37.31	36.35	35.41	34.50	33.61	32.75	31.90	31.08	30.28	29.50	28.74	28.00
Plateau	59.23	59.20	59.18	59.16	59.13	59.11	59.08	59.06	59.03	59.01	58.99	58.96
Zou	35.85	34.35	32.91	31.53	30.21	28.95	27.74	26.57	25.46	24.40	23.37	22.39
**Benin**	52.85	52.16	51.47	50.80	50.13	49.48	48.83	48.19	47.55	46.93	46.31	45.71
