# Supplementary material for: Is Benin on track to reach universal household coverage of basic water, sanitation and hygiene services by 2030?
Source: PLoS One. 2023 May 25;18(5):e0286147. doi: 10.1371/journal.pone.0286147 (PMC10212078; doi:10.1371/journal.pone.0286147)
Supplement: S17 Table — (PDF) [file pone.0286147.s017.pdf]

S17 Table. Projections of household access to basic hygiene services, Benin, 2019-2030

**Variables**	**Projections (%)**											
	**2019**	**2020**	**2021**	**2022**	**2023**	**2024**	**2025**	**2026**	**2027**	**2028**	**2029**	**2030**
**Age (years)**												
<30	11.93	13.68	15.68	17.98	20.62	23.64	27.11	31.08	35.64	40.86	46.85	53.72
30-39	11.42	12.50	13.68	14.97	16.38	17.92	19.61	21.46	23.48	25.70	28.12	30.77
40-49	12.05	12.86	13.73	14.65	15.64	16.70	17.82	19.02	20.31	21.68	23.14	24.70
50-59	12.41	13.52	14.72	16.03	17.46	19.01	20.71	22.55	24.56	26.74	29.12	31.71
≥60	10.99	12.43	14.05	15.89	17.97	20.33	22.99	26.00	29.40	33.25	37.60	42.53
**Sex**												
Male	11.80	12.99	14.31	15.76	17.35	19.11	21.04	23.18	25.52	28.11	30.95	34.09
Female	11.20	12.23	13.36	14.59	15.93	17.40	19.00	20.75	22.66	24.75	27.03	29.52
**Level of education**												
No formal education	8.29	10.34	12.90	16.09	20.07	25.04	31.23	38.96	48.60	60.62	75.61	94.32
Primary	11.25	12.70	14.33	16.17	18.24	20.59	23.23	26.22	29.58	33.39	37.67	42.51
Secondary	17.07	18.25	19.52	20.86	22.31	23.85	25.50	27.26	29.14	31.16	33.31	35.62
Higher	33.25	33.30	33.35	33.40	33.45	33.49	33.54	33.59	33.64	33.69	33.74	33.79
**Marital status**												
Single	12.25	13.66	15.23	16.98	18.93	21.11	23.54	26.25	29.26	32.63	36.38	40.57
In couple	12.49	14.46	16.75	19.39	22.46	26.00	30.11	34.87	40.38	46.75	54.14	62.69
**Wealth index**												
Poorest	5.71	7.46	9.76	12.76	16.68	21.81	28.51	37.28	48.74	63.72	83.31	>99.00
Poorer	8.81	12.11	16.65	22.89	31.46	43.24	59.43	81.69	>99.00	>99.00	>99.00	>99.00
Middle	9.32	11.97	15.36	19.72	25.31	32.48	41.69	53.52	68.69	88.17	>99.00	>99.00
Richer	12.09	15.15	18.97	23.76	29.76	37.27	46.68	58.46	73.22	91.70	>99.00	>99.00
Richest	26.65	29.09	31.75	34.65	37.81	41.27	45.05	49.16	53.66	58.56	63.92	69.76
**Household size**												
≤5	12.22	13.46	14.82	16.32	17.98	19.80	21.80	24.01	26.44	29.12	32.07	35.31
>5	10.73	11.75	12.88	14.11	15.46	16.94	18.55	20.33	22.27	24.40	26.73	29.29
**CU5 in the household**												
No	12.44	13.39	14.41	15.51	16.70	17.97	19.35	20.82	22.42	24.13	25.97	27.96
Yes	11.31	12.76	14.40	16.25	18.34	20.70	23.36	26.36	29.75	33.57	37.88	42.74
**Area**												
Urban	15.05	15.95	16.91	17.93	19.01	20.15	21.36	22.65	24.01	25.46	26.99	28.61
Rural	9.90	12.11	14.81	18.12	22.16	27.10	33.14	40.53	49.57	60.62	74.14	90.67
**Department**												
Alibori	10.96	13.24	16.00	19.34	23.36	28.23	34.11	41.22	49.80	60.18	72.71	87.86
Atacora	4.91	5.52	6.21	6.99	7.86	8.84	9.95	11.19	12.60	14.17	15.94	17.94
Atlantique	20.16	21.55	23.04	24.64	26.34	28.16	30.11	32.19	34.42	36.80	39.34	42.06
Borgou	16.34	20.18	24.93	30.80	38.05	47.01	58.08	71.75	88.63	>99.00	>99.00	>99.00
Collines	18.43	22.40	27.23	33.10	40.23	48.90	59.43	72.24	87.81	>99.00	>99.00	>99.00
Couffo	12.40	14.55	17.08	20.04	23.51	27.58	32.37	37.98	44.56	52.28	61.34	71.97
Donga	6.77	7.76	8.89	10.18	11.66	13.35	15.30	17.52	20.07	22.99	26.33	30.16
Littoral	33.23	36.53	40.15	44.14	48.52	53.33	58.62	64.44	70.84	77.87	85.60	94.09
Mono	5.11	5.71	6.38	7.12	7.96	8.89	9.92	11.08	12.38	13.83	15.45	17.25
Ouémé	8.30	8.93	9.61	10.34	11.12	11.97	12.87	13.85	14.90	16.04	17.25	18.57
Plateau	1.45	1.42	1.39	1.35	1.32	1.29	1.26	1.23	1.20	1.17	1.15	1.12
Zou	4.59	5.16	5.81	6.54	7.36	8.28	9.31	10.48	11.79	13.26	14.92	16.79
**Benin**	11.65	12.81	14.08	15.47	17.01	18.69	20.55	22.59	24.83	27.29	30.00	32.98
