# Supplementary material for: Is Benin on track to reach universal household coverage of basic water, sanitation and hygiene services by 2030?
Source: PLoS One. 2023 May 25;18(5):e0286147. doi: 10.1371/journal.pone.0286147 (PMC10212078; doi:10.1371/journal.pone.0286147)
Supplement: S1 Fig — (PDF) [file pone.0286147.s018.pdf]

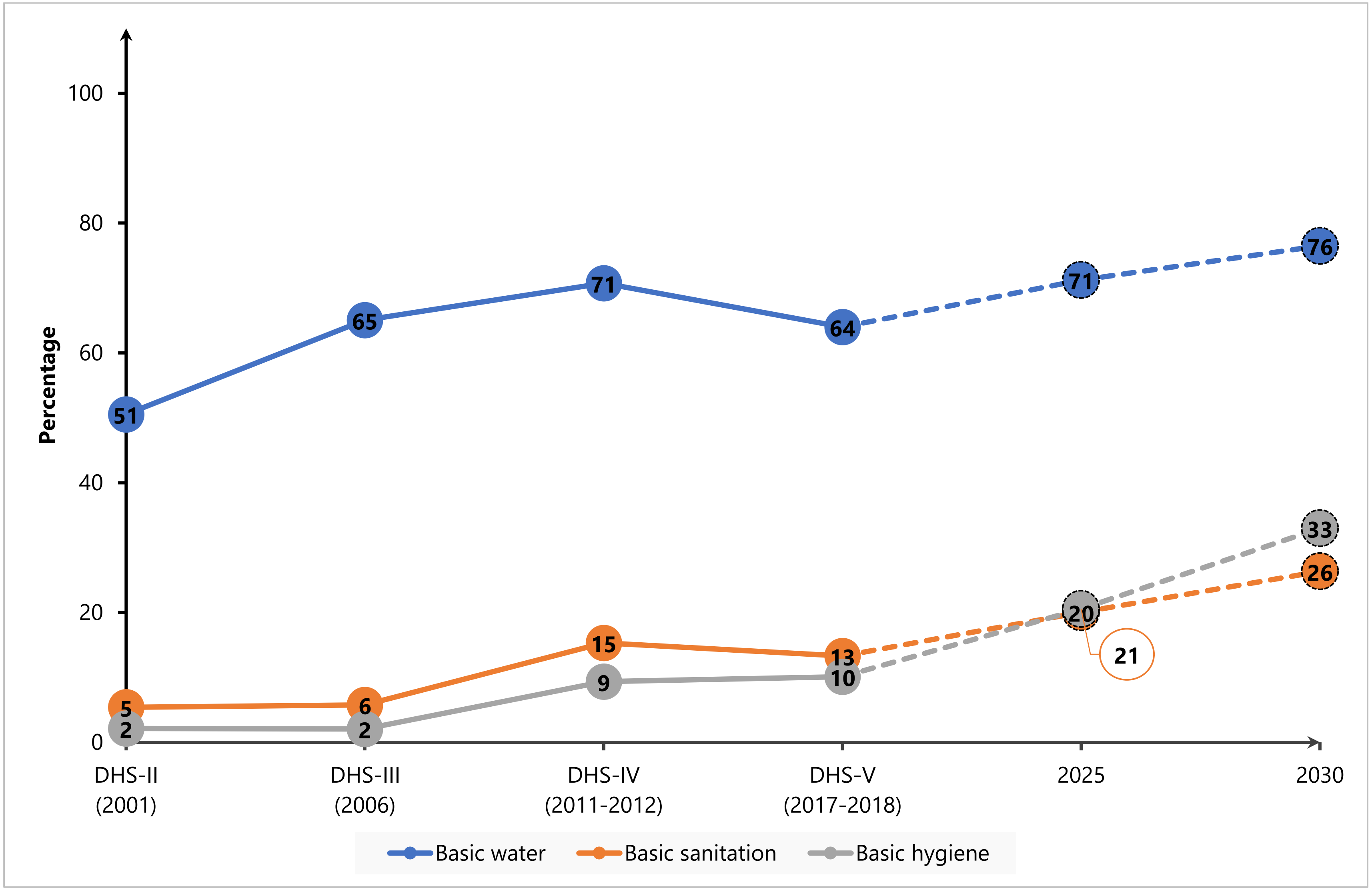

S1 Fig. Evolution of household access to individual basic WASH services from 2001 to 2017-2018, and projection to 2030, Benin
