# Supplementary material for: Is Benin on track to reach universal household coverage of basic water, sanitation and hygiene services by 2030?
Source: PLoS One. 2023 May 25;18(5):e0286147. doi: 10.1371/journal.pone.0286147 (PMC10212078; doi:10.1371/journal.pone.0286147)
Supplement: Attachment — Submitted filename: attachment_reviewPONE-D-22-29273.docx [file pone.0286147.s020.docx]

**Manuscript Number	:** PONE-D-22-29273

**Manuscript Title :** Is Benin on track to reach universal household coverage of basic water, sanitation and hygiene services by 2030?

Thank you for invite me review this paper.

In general, this paper is well-written, interesting and useful. This can be evidence for monitoring inequity. The paper is well-planned and easy to read.

The methodology appears sound, but I have made suggestions below that would improve or clarify the findings and some inputs for other parts.

1. **Abstract:**

The objective state 'make projection for 2030', but in conclusion, the Author has not clearly stated the projection result. The Author needs to add a statement to improve the conclusion.

2. **Methods:**
a. Sampling (line 151), I feel like there is missing information at ‘each department’. Add words/sentence that can connect with each department.
b. In the introduction section, the authors have explained the differences in standard definitions of the WASH indicators in the MDGs and SDGs. The Author added reference information about the standard definition of WASH in table 1.
c. Table 1: The Author needs to add an explanation of how to create variables 'improved water' and 'improved sanitation' according to Benin DHS and references
d. Table 1 (lines 196-197): did this study conduct reanalyses following the operational definition written in table 1?
e. In lines 195-198, the Author needs to add information describing tables S1 and S2.
f. Table S3, mention p value, is there a subgroup as reference?
3. **Results:**
a. Benin DHS, which were carried out based on a sample with complex design. It is better to present the data displays with a 95% confidence interval.
b. In line 243, the Author needs to add the meaning of the p-value in tables S1 and S2.
c. Line 264; although it has summarized tables S8-S12, it would be more communicative to add the word ‘Table S3’ before ‘the overall’ (line 265).
d. Line 270; what is the meaning of minus value? The Author needs to give a brief explanation.
e. Projection of access to WASH by 2030, add number of table that is being discussed in the text of the result.
4. **Discussion**
a. Lines 311-315 are the justification of the study. I suggest these four sentences move to the end of the introduction.
b. Line 318, add '**(Table S2)**' after 63,68% to clarify this.
c. Line 319-320 – which table was referred to? State the number of tables
d. The SDGs' slogan is no one leave behind and interconnection between SDGs’ goals. This paper will be interesting if it discusses connection with the goal#10- inequity that affects the achievement of the 2030 target in Benin. This paper can be evidence-based for monitoring inequity in Benin when discussing the characteristics of being able to raise which subgroups are disadvantaged so that interventions can address these sub groups with the proper programs to reduce the gap between subgroups. Then these will increase the achievement of WASH targets.
e. The Author can add discussion abour policy recommendations for Benin's government based on this study.
5. **Conclusion**

Rewrite more clearly to answer the research question related to the projection result.

6. **Others**
a. Tables; make the title of the table and text. Example text: 'Level of household access to water' title of table S3: 'access to basic drinking water services'.
   I suggest making it consistent.
b. Table presentation – to be self-reported. Add a footnote about the p-value – what you want to explain from the p-value so that the table is easier to read.
c. In each table, add in lowest row for total Benin, ‘**n’** and ‘**%’** in each column
d. In table S1-S7, it is better to add the year after DHS II ‘(**2001**)’, DHS III ‘(**2006**)’, DHS IV ‘(**2011-2012**)’, DHS V ‘(**2017-2018**)’
